# Adverse birth outcome and associated factors among mothers with HIV who gave birth in northwest Amhara region referral hospitals, northwest Ethiopia, 2020

**DOI:** 10.1038/s41598-022-27073-2

**Published:** 2022-12-29

**Authors:** Elsa Awoke Fentie, Hedija Yenus Yeshita, Ever Siyoum Shewarega, Moges Muluneh Boke, Attitegeb Abera Kidie, Tewodros Getaneh Alemu

**Affiliations:** 1 Department of Reproductive Health, Institute of Public Health, University of Gondar, Po. Box: 196, Gondar, Ethiopia; 2 Department of Pediatric Nurse, School of Nursing, University of Gondar, Gondar, Ethiopia; 3 Department of Public Health, College of Health Science, Woldia University, Woldia, Ethiopia

**Keywords:** Immunology, Health care

## Abstract

Adverse birth outcomes are a common cause of health problems in developing nations and have a significant negative impact on infant health as well as financial costs to families, communities, and the world. Mothers with HIV may be at increased risk of adverse birth outcomes. However, there is a limited study about adverse birth outcomes among mothers with HIV around the world including in Ethiopia. Therefore this study aimed to assess adverse birth outcomes and associated factors among mothers with HIV Facility based cross-sectional study was conducted among mothers with HIV who gave birth in northwest Amhara region referral hospitals from September 2016 to September 2019. Simple random sampling was used to select 590 mothers. Bivariable and multivariable logistic regressions were carried out to identify factors. Statistical significance was declared by using a p-value < 0.05. An adjusted odds ratio was used to show the magnitude of the association. Out of a total of 590 mothers, the prevalence of adverse birth outcomes among HIV-positive mothers was 21% (95% CI 17.8–24.6%). History of spontaneous abortion [AOR = 1.9, 95% CI (1.19, 3.70)], PROM [AOR = 3.55, 95% CI (1.72, 7.30)], opportunistic infection [AOR = 3.38, 95% CI (1.50, 8.22)], pre-pregnancy BMI of < 18.5 [AOR = 5.61, 95% CI (1.97, 15.91)], MUAC < 23 cm [AOR = 2.56, 95% CI (1.10, 5.97)], and ANC visit of < 4 times [AOR = 3.85, 95% CI (2.34, 6.55)] were significantly associated with Adverse birth outcome. The prevalence of adverse birth outcomes was high. Abortion history, MUAC, BMI, Opportunistic infection, PROM, and a number of ANC visits were associated with adverse birth outcomes. This study suggests to increase number of antenatal care follow-ups, prevent and treat opportunistic infections, and focus on early detection and treatment of pregnancy-related complication

## Introduction

HIV continues to be a burden on women and children worldwide^1^. In 2019, 37.9 million people were living with HIV^2^. The sub-Saharan region is the most affected place in the world with 25.6 million people living with HIV^3^. In Ethiopia by 2018, nearly 690,000 people were living with HIV/AIDS and 11,000 people have died from AIDS^4^. Although HIV infection has been reported to have little effect on pregnancy outcomes in the developed world, early studies from sub-Saharan Africa suggest that infants of mothers with HIV may be at increased risk of adverse pregnancy outcomes such as lower birth weight, prematurity, and perinatal and neonatal death^5,6^.

Each year, 15 million babies are preterm birth around the world^7^ and preterm birth is the leading cause of neonatal deaths which is contributing to 35% of the world’s neonatal mortality^8^. Globally 20.5 million live births is suffered from LBW in 2015 almost half of them in Southern Asia which is 9.8 million and about one-quarter of all LBW newborns are in sub-Saharan Africa^9^. Worldwide, over 2.6 million deliveries are stillbirths 98% of which occur in low and middle-income countries; 77% in South Asia and sub-Saharan Africa^10^.

Adverse birth outcomes are a common cause of health problems in developing nations and have a significant negative impact on infant health as well as emotional and financial costs to families, communities, and the world, especially in resource-constrained settings with underdeveloped health systems and weak access to and use of health services^11^. Children born with low birth weight and preterm are more likely to die prematurely compared to infants of normal birth weight and gestational age^12^. Likewise, these children experience more morbidity, both in the short and long term. Among these respiratory distress, heart problems, chronic lung disorders, growth impairment, blindness or low vision, deafness, cognitive impairment, and cerebral palsy are the problems associated with low birth weight and preterm babies^13,14^. There is also a greater vulnerability to infectious diseases due to a poorer immune response^15^.

Studies conducted in different areas reported risk factors for Adverse birth outcomes: low socio-economic status, MUAC < 23 cm, pregnancy-related complications and history of preterm birth, age less than 20, PROM, UTI, multiple pregnancies, preeclampsia, fetal malformation, polyhydramnios, antepartum hemorrhage, previous abortion, residence, lack of antenatal care, maternal disease, null parity, The presence of chronic illness, the absence of antenatal follow-up, HIV status of the mother, CD4 count, Viral load, WHO clinical stage, and anemia were found to be significantly associated with Adverse birth outcomes^16–21^.

In Ethiopia, globally recommended effective strategies have been implemented for a newborn with an adverse birth outcome with given emphasis on the packages of care provided at the prenatal, ante-natal, intrapartum, and postnatal periods. However, the outcome during pregnancy and delivery periods is a major challenge in realizing the seated goal of Sustainable Development Goals. While the effect of HIV infection on maternal morbidity, mortality, and vertical transmission to her offspring is well established. But there is a limited study about adverse birth outcomes among mothers with HIV in Ethiopia. Therefore, this study aims to assess adverse birth outcomes and associated factors among HIV-positive mothers.

## Methodology

### Study design and period

An institutional-based cross-sectional study was conducted among mothers delivered from September 2016 to September 2019 in northwest Amhara region referral hospitals and the data was extracted from March 3 to May 18/2020.

### Study area

Amhara national region is one of the ten national states in Ethiopia which is found in the Northern part of Ethiopia. The region has 80 hospitals, 847 health centers, and 3342 health posts. there are 6 referral hospitals—namely Gondar University Comprehensive Specialized Hospital (GUCSH), Felegehiwot Comprehensive Specialized Hospital (FCSH), Dessie Referral Hospital (DRH), Debre-Markos Referral Hospital (DMRH), Debre-Birhan Referral Hospital (DBRH) and Debre tabor referral hospitals. Three out of six referral hospitals were found in the Northwest part of the Amhara region. These include: —the University of Gondar comprehensive and specialized Hospital (UoGCSH), Felege Hiwot comprehensive, and specialized hospital (FHCS), and Debre Markos referral hospital. Each referral hospital’s catchment population is estimated to be 5–7 million people. The annual average number of births in each hospital is 6000 per year. according to the hospital report. All three hospitals are providing full ANC/PMTCT, ART, delivery services, and ultrasound-guided obstetric care.

### Eligibility criteria

All mothers with HIV delivered from September 2016–September 2019 in northwest Amhara region referral hospitals with a gestational age of 28 weeks and above were included in the study. However, mothers who had unknown or unreliable last normal menstrual period (LNMP) with the absence of ultrasound evidence and a mother with unrecorded birth outcome were excluded from the study.

### Variables of the study

The dependent variable in this study was the adverse birth outcome. Whereas Socio-demographic, obstetric, medical, nutritional, and HIV-related variables were the independent variables. This includes age, residence, educational status, marital status history of substance use, including alcohol drinking and smoking, nutritional counseling during ANC, iron and folic acid supplementation during pregnancy, pre-pregnancy BMI, MUAC, CD4 count, viral load, WHO clinical stage of the disease, initiation of ART, time of initiation of ART, time of diagnosis with HIV, types of ARV, anemia, chronic medical disease, Urinary tract infection (UTI), pregnancy-induced hypertension (PIH), Antepartum hemorrhage (APH), the premature rapture of the membrane (PROM), previous history of abortion, previous history of stillbirth, parity, and gravidity.

### Operational definition

Adverse birth outcomes: A woman who had at least one of the following stillbirth, low birth weight, preterm birth^22^.

Preterm birth: Preterm is defined as babies born alive before 37 weeks of gestation but after viability (28 weeks of gestation) and gestational age was calculated based on LNMP or first-trimester ultrasound result^23^.

Low birth weight: a birth weight < 2500 g irrespective of gestational age^24^.

Stillbirth*:* dead birth after the 28th week of gestation and before the expulsion from the uterus^25^.

APH: defined as any vaginal bleeding in the mother after 28 weeks of gestation as documented in the records by the attending clinician^26^.

PIH: defined clinically as a blood pressure of > 140/90 mmHg after 20 weeks of gestation with or without proteinuria and/or edema as diagnosed and documented by the attending clinician^26^.

Anemia: documented Hgb level below 11gm/dl laboratory diagnosis^26^.

UTI: Defined as a documented clinical/laboratory diagnosis of UTI at any time during the pregnancy^26^.

### Sample size determination and sampling procedure

**Figure 1:**
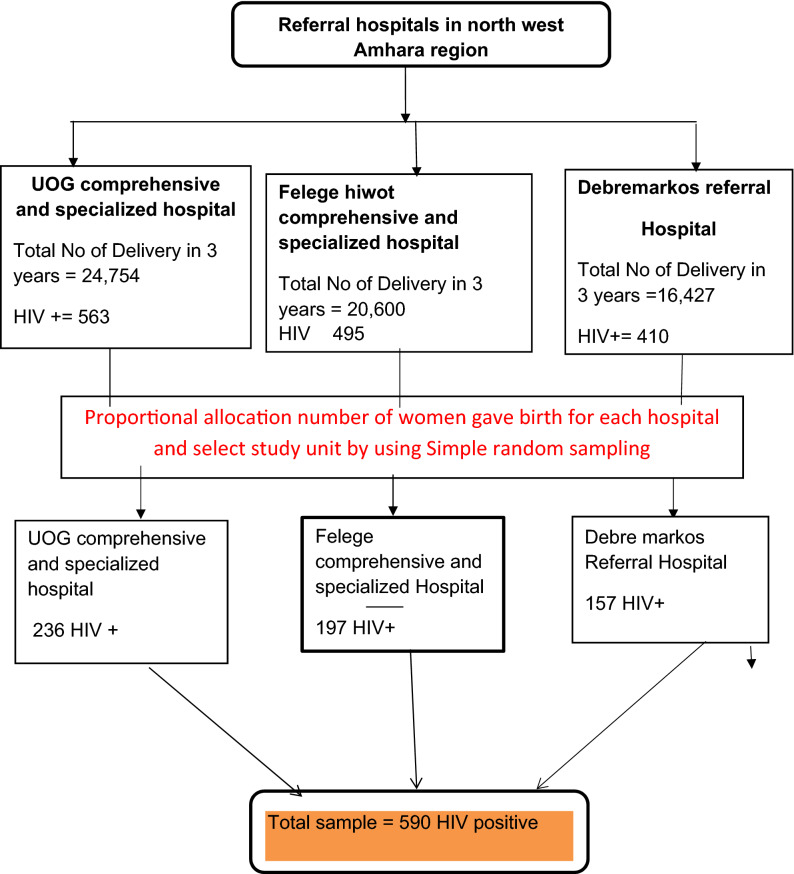
Schematic presentation of the sampling procedure for the prevalence of an adverse birth outcome and associated factors among mothers with HIV-positive delivered in northwest Amhara region referral hospitals.

The required sample size was determined by using the single population proportion formula n = za^2^/2p(1 − p)/d^2^ by considering the prevalence PTB among mothers with HIV was 16.6%^16^, 95% confidence interval (CI), 3% margin of error to yield a total of 590 study participants. The total sample size was proportionally allocated for the three Hospitals depending on their load of delivery. A simple random sampling technique was employed to select the study participant’s medical records. The delivery registration logbooks were used as a sampling frame and selected each record for our study used a computer-generated random number. Whenever the selected chart did not fulfill the inclusion criteria, the next medical record was considered (Fig. 1.).

### Data collection tool

The patient's medical records were used as a source of data. The data, consisting of socio-demographic variables, clinical and obstetric history as well as birth outcome, were collected using a data extraction tool. Maternal BMI was determined by using the mother’s pre-pregnancy, initial weight, and height from their ART and PMTCT follow-up. Newborn weight was measured using standard beam balance within the first hour of birth Six Bachelor of science (BSc) Midwives collected the data, while 3 midwives who have a second degree in clinical midwifery supervised the data collection process. Data quality was maintained by the following data quality control mechanisms; A 5% preliminary chart review was conducted in the Gondar university comprehensive and specialized hospital before the actual data collection and amendments were considered based on the result of a preliminary chart review. One day of training was given to data collectors and supervisors. Strict supervision of the data collection was carried out throughout the data collection period. The collected data was checked for its consistency and completeness before any attempt to enter, code, and analyze it.

### Data processing and analysis

Data were coded and then entered using EPI data version 4.6 and exported to SPSS. The final statistical analysis was done by SPSS version 25. Before analysis, data were cleaned using frequency; listing, and sorting to identify any missed values, and then corrections were made by revising the original questionnaire. There are different techniques of missing data management. Deletion, replacement using the mean or mode of the data (mean substitution/replacement) or predicted values from a regression to substitute the missing values. So for this study, we had used replacement by mean for continuous and Mode for categorical variable if less than 20% of values are missed in one variable, but if more than 20% of values are missed in one variable, we discard the variables. Descriptive statistics were made for categorical variables using frequencies. The result was presented using texts and tables. a multivariable binary logistic regression model was used to assess the association between dependent and independent variables. P-value < 0.05 and Adjusted Odds Ratio (AOR) with 95% CI was used to declare statistically significant predictors in multivariable analysis.

### Ethical approval and informed consent of participants

Ethical approval was obtained and the need for informed consent was waived by the ethical review committee of the Institute of public health on behalf of the Institutional Review Board (IRB) of the University of Gondar. Permission was obtained from the clinical director of each hospital. Since this study uses secondary data to ensure confidentiality Personal Identifiers Were not used on the data collection form, and all data were kept strictly confidential and used only for the study purposes. The research was conducted according to the Helsinki declarations.

## Result

### Socio-demographic and HIV-related characteristics of mothers

Regarding sociodemographic variable such as: educational status and occupational status had missing values of 30 and 43 respectively. These missing values replaced by their Modal value. The study result showed that the majority 564 (95.6%) of respondents were from an urban area and the rest were from a rural area. Regarding the religion of respondents; the majority 553 (93.7%) were orthodox followers and the rest were Muslim and protestant followers. Almost all 584 (99%) mothers with HIV received ARV intervention. Most (84.4%) mothers started their HAART before pregnancy. Regarding their adherence, almost all 578 (98%) have good adherence (Table 1).

**Table 1:** Socio-demographic and HIV-related characteristics of mothers delivered in west Amhara regional state referral hospitals (N = 590).

Variables	Category	Frequency	%
Age category	17–24	58	9.8
	25–34	411	69.7
	≥ 35	121	20.5
Residence	Rural	26	4.4
	Urban	564	95.6
Educational status	Uneducated	174	29.5
	Primary school	148	25.1
	Secondary and above	268	45.4
Marital status	Married	549	93
	Unmarried	41	7
Time of HIV diagnosis	Before pregnancy	488	82.7
	During pregnancy and delivery	102	17.3
Time to start HAART	Before pregnancy	493	84.4
	During pregnancy	91	15.6
Regimen	1c	166	28.4
	1d	26	4.5
	1e	352	60.3
	Other	40	6.8
CD4 count	< 200	480	81.4
	200–350	86	14.6
	≥ 351	24	4
Viral load	TND	545	92.4
	< 1000	34	5.8
	≥ 1000	11	1.8

*TND; target not detected, *HAART; highly active antiretroviral therapy, *1c; AZT + 3TC + NVP, *1d; AZT + 3TC + EFV, *1e; TDF + 3TC + EFV.

### Nutrition and medical-related characteristics of mothers

Regarding nutrition variable such as: pre pregnancy BMI and MUAC had missing values of 25 and 14 respectively. These missing values replaced by the mean value. Almost all 583 (98.81%) respondents had taken iron/folate during pregnancy Among those who took iron/folate during pregnancy, 359 (61.58%) of mothers taken with a duration of fewer than 3 months. Almost all 583 (98.81%) respondents received nutritional counseling during their ANC visit. A majority, 524 (88.81%) and 547 (92.71%), of mothers, had MUAC ≥ 23 cm and pre-pregnancy BMI of ≥ 18.5 respectively. A few proportions 36 (6.10%) of mothers are anemic and the rest/majority 554 (93.90%) of them had no anemia. Regarding medical conditions, about 31 (5.25%) had STI and 43 (7.29%) had UTI.

### Pregnancy and labor-related complications of the mothers

Almost all 585 (99.15) mothers with HIV have ANC follow-ups. Only 27 (4.58%) of mothers had prolonged labor the rest/majority of them don’t have prolonged labor (Table 2).

**Table 2:** pregnancy and labor-related complications of mothers with HIV delivered in west Amhara regional state referral hospitals (N = 590).

Variables	Category	Frequency	%
Gravidity	Primi gravidia	97	16.4
	Multi gravida	493	83.6
Parity	Null parity	120	20.3
	Primiparity	192	32.5
	Multiparity	278	47.1
Abortion	No	508	86.1
	Yes	82	13.9
Number of ANC visits	≥ 4	372	63.6
	< 4	213	36.4
Pregnancy status	Planned and wanted	427	72.4
	Unplanned but wanted	154	26.1
	Unplanned and unwanted	9	1.5
APH	No	580	98.3
	Yes	10	1.7
PROM	No	543	92.0
	Yes	47	8.0
PIH	No	562	95.2
	Yes	28	4.8
Labor status	Induced	41	7.0
	Spontaneous	511	86.6
	Elective C/S	38	6.4
Mode of delivery	SVD	457	77.5
	Caesarian section	127	21.5
	Instrumental delivery	6	1.0

### Prevalence of adverse birth outcome

This study's finding showed that the prevalence of adverse birth outcomes among HIV-positive mothers was 21.4% (95% CI 18–25%). Out of 590 births 10 (1.7%) were stillbirth, 98 (16.6%) were LBW, 74 (12.5%) were preterm, 5 (0.8%) were both stillbirth and LBW, 50 (8.5%) LBW and preterm, 4 (0.68%) Still birth and preterm, and 4 (0.68%) were Still birth, LBW and preterm.

### Determinants of adverse birth outcome

In multivariable analysis; history of spontaneous abortion, PROM, presence of opportunistic infection, BMI, MUAC, and ANC visit was significantly associated with adverse birth outcome. This study result showed that having a history of spontaneous abortion increases the risk of adverse birth outcomes by 1.9 times [AOR = 1.9, 95% CI (1.19, 3.70)]. The odds of adverse birth outcomes among mothers who had PROM during current pregnancy were 3.55 times [AOR = 3.55, 95% CI (1.72, 7.30)] higher as compared with their counterparts and other variables kept constant. Regarding opportunistic infection; HIV positive mothers with opportunistic infection were 3.38 times [AOR = 3.38, 95% CI (1.50, 8.22)] more likely to develop adverse birth outcomes as compared to those who don’t have an opportunistic infection and the effect of other variables remained constant. Those HIV-positive mothers with pre-pregnancy BMI of < 18.5 were 5.61 times [AOR = 5.61, 95% CI (1.97, 15.99)] more likely to have an adverse birth outcome as compared with their counterparts.

The study result showed that a mother with MUAC < 23 cm increases the risk of adverse birth outcome by 2.56 times [AOR = 2.56, 95% CI (1.10, 5.97)] as compared to those with MUAC ≥ 23 cm and other variables remained constant. Regarding ANC visits; mothers with ANC visits of < 4 times were 3.85 times [AOR = 3.85, 95% CI (2.34, 6.55)] more likely to have adverse birth outcomes as compared to mothers with ≥ 4 ANC visits (Table 3).

**Table 3:** Factors associated with adverse birth outcome among mothers with HIV-

Variables	Category	Adverse birth outcome		AOR (95% CI)
		No	Yes	
Age category	17–24	48	10	1
	25–34	323	88	1.43 [0.58, 3.53]
	≥ 35	93	28	1.97 [0.69, 5.65]
Residence	Rural	18	8	1
	Urban	446	118	0.45 [0.16, 1.29]
Educational status	Uneducated	137	37	1
	Primary school	119	29	1.49 [0.75, 2.95]
	Secondary and above	208	60	1.63 [0.89, 2.97]
Marital status	Married	430	119	1
	Unmarried	34	7	0.59 [0.21, 1.64]
Parity	Null parity	91	29	1
	Primiparity	152	40	0.52 [0.17, 1.59]
	Multiparity	221	57	0.48 [0.15,1.51]
Time of HIV diagnosis	Before pregnancy	387	101	1
	During pregnancy and delivery	77	25	1.69 [0.85, 3.03]
Abortion	No	409	99	1
	Yes	55	27	1.9 (1.19, 3.70)*
PROM	No	439	104	1
	Yes	25	22	3.55 (1.72, 7.30)*
CD4 count	≥ 351	391	90	1
	200–350	60	26	2.23 (0.87, 4.11)
	< 200	13	10	2.24 (0.73, 6.91)
Viral load	TND	433	112	1
	< 1000	23	11	1.03 [0.39, 2.68]
	≥ 1000	8	3	0.74 [0.14, 4.01]
Presence of OI	No	447	114	1
	Yes	17	12	3.38 (1.50, 8.22)*
BMI	≥ 18.5	450	97	1
	< 18.5	14	29	5.61 (1.97, 15.91)*
MUAC	≥ 23	436	88	1
	< 23	28	38	2.56 (1.10, 5.97)*
Duration of iron intake	≥ 3 month	202	22	1
	< 3 month	257	102	1.67 (0.88, 3.17)
Anemia	No	445	109	1
	Yes	19	17	2.29 (0.95, 5.51)
Number of ANC visit	≥ 4	328	44	1
	< 4	132	81	3.80 (2.34, 6.55)*

*p < 0.005.

## Discussion

The finding of the study showed that the prevalence of adverse birth outcomes was 126 (21.4% 95% CI 18–25%) among which 10 (1.7%) were stillbirth, 74 (12.5%) were preterm and 98(16.6%) were low birth weight. These figures were comparable with findings from studies conducted in Hosanna (24.5%)^13^, Hawassa (18.3%), Tanzania (18%), and Ghana (19%) of mothers who experienced adverse birth outcomes^27,28^.

The overall prevalence of adverse birth outcomes in this study was slightly lower than in studies conducted in Ethiopia, Dessie, 32.5%^24^ The discrepancy might be due to a difference in the study area and study participant's residents and age group. the study conducted in Dessie used 30–40% of rural resident participants and rural resident mothers are highly prone to adverse birth outcome than those living in urban^29^ and 15% of study participants in Dessie was Age group < 20 years and this age group is highly prone adverse birth outcome^13,30^.

The overall prevalence of adverse birth outcomes in this study is slightly higher than in a study done in Kembata (13.9%)^25^ this might be due to the difference in the study area, this study is done in referral hospitals whereas a study done in Kembata were in a health center and primary hospital. This may be because most normal deliveries take place in health centers while more complicated ones are referred to the tertiary hospital contributing to higher rates of adverse birth outcomes at referral hospitals^20^ and study participants in this study were HIV positive and HIV increases the risk of having adverse birth outcome^23,31^ or it might be due to compromised immune system of HIV positive mothers increase the risk of opportunistic infection, which contributes to the occurrence of adverse birth outcome.

Mothers who had opportunistic infections during pregnancy were found to be more than 3 times more likely to have Adverse birth outcomes than mothers who did not have Opportunistic infections. this finding is consistent with studies done in Nigeria^32^. This might be due to opportunistic infections compromising the nutritional status of the mother and fetal growth, for instance, infants from mothers with syphilis are often premature^33^.

In HIV-positive women the risk of having adverse birth outcomes were more than fourfold higher in a mother who had pre-pregnancy BMI less than 18.5 when compared with mothers having pre-pregnancy BMI ≥ 18.5. This result was similar to a study done in northwest Ethiopia public hospitals^16^. Moreover, In these findings mothers with MUAC, less than 23 cm were also found to experience adverse birth outcomes when compared with those with MUAC greater than 23 cm this result is in agreement with the study done in Dessie referral hospital^20^ This might be due to intergenerational effect of malnutrition that leads to LBW^29^ or poor nutritional status of the mother compromised the immune system and increase the risk of opportunistic infections that leads to preterm birth.

In this study, mothers with less than four times ANC visits were 3.5 times at high risk to have Adverse birth outcomes than those with four and more visits. This result was similar with study done in Mekell Hospital^30^ Debre tabor^23^, Jima^34^, Dodola town^35^, and Metu^36^. This might be due to the lack of adequate ANC visits decreases the chance of identifying risks factors of Adverse birth outcome early and providing appropriate interventions^23^.

Pregnancy-related complication during the current pregnancy was significantly associated with Adverse birth outcome, according to the finding of this research the risk of having adverse birth outcome among mothers who had PROM during the current pregnancy were more than 3 times higher than those mothers who had no PROM. This finding is consistent with a study done Debre tabor^23^, East Africa^37^, Ghana and Kenyatta national hospital^38,39^, Axum and Adwa public hospitals^40^, and Hosanna^13^. This might be due to that labor will spontaneously initiate within a week after preterm PROM^23^, or this might be due to -provider-initiated early termination of pregnancy to manage pregnancy-related complications^20^.

## Limitation of study

Since this study is hospital-based, it doesn’t include mothers who gave birth at home, which makes it difficult to conclude the general population. In addition, the data used were secondary there may be bias and incomplete information or missing variables.

## Data Availability

Data will be available upon request from the corresponding author.

## Abbreviations

AIDS: Acquire immune deficiency syndrome
ANC: Antenatal care
AOR: Adjusted odd ratio
APH: Anti partum hemorrhage
ART: Anti-retroviral treatment
BMI: Body mass index
CI: Confidence interval
COR: Crude odds ratio
DM: Diabetes mellitus
HAART: Highly active anti-retroviral therapy
HIV: Human immune virus
HTN: Hypertension
LBW: Low birth weight
LNMP: Last normal menstrual period
MUAC: Mid upper arm circumference
PIH: Pregnancy induced hypertension
PMTCT: Prevention of mother to child transmission
PROM: Premature rupture of membrane
PTB: Pre term birth
SD: Standard deviation
SPSS: Statistical package for social science
UOG: University of Gondar
UTI: Urinary tract infection
WHO: World Health Organization
